# Smokers and Non-Smokers in the Polytechnic School of Paris

**Published:** 1860-12

**Authors:** 


☞ In the September number of the London *Pharmaceutic Journal* for 1860, it is stated that, on dividing the pupils of the Polytechnic School of Paris into smokers and non-smokers, it is shown that the non-smokers have proved themselves, in the various competitive examinations on entering the schools, scholars of a lower rank; but in the various ordealsthey have to pass through in a year, the average rank of the smokers has constantly fallen, and not inconsiderably, when the men who did not smoke enjoyed a cerebral atmosphere of the clearest kind.

